# Comment on “Effectiveness of Mind–Body Exercise in Older Adults With Sarcopenia and Frailty: A Systematic Review and Meta‐Analysis” by Wan et al.

**DOI:** 10.1002/jcsm.13856

**Published:** 2025-06-04

**Authors:** Alejandro Álvarez‐Bustos, Leocadio Rodriguez‐Mañas, Emanuele Marzetti, Hélio José Coelho‐Júnior

**Affiliations:** ^1^ Biomedical Research Center Network for Frailty and Healthy Ageing (CIBERFES) Institute of Health Carlos III Madrid Spain; ^2^ Instituto de Investigación IdiPaz Madrid Spain; ^3^ Department of Geriatrics Hospital Universitario de Getafe Madrid Spain; ^4^ Fondazione Policlinico Universitario Agostino Gemelli IRCCS Rome Italy; ^5^ Department of Geriatrics and Orthopedics Università Cattolica del Sacro Cuore Rome Italy

We read with interest the recent article by Wan and colleagues, entitled ‘Effectiveness of Mind–Body Exercise in Older Adults With Sarcopenia and Frailty: A Systematic Review and Meta‐Analysis’ [1]. The article examined the effects of mind–body exercise (MBE) on mitigating sarcopenia and frailty in older adults. The topic is of high relevance, as both frailty and sarcopenia are prevalent conditions in the older population and known to increase the risk of several negative health events. As the authors correctly suggest, these conditions may be reversible through targeted interventions. That said, we believe the article raises several points that merit discussion to allow a more comprehensive interpretation of the results.

Firstly, the authors introduce their work by questioning the suitability of high‐load resistance training programmes for older adults, *quod*: ‘such programs may *cause excessive mechanical* stress due to the *reduced number of muscle fibers available*, *potentially increasing the risk of injury*’. This argument is fundamentally flawed and could potentially discourage the prescription of resistance training in this population.

Indeed, high‐quality randomized clinical trials involving the performance of progressive and supervised high‐load resistance training programmes have not reported the occurrence of clinically relevant pain or injuries in function of exercise, even in highly vulnerable individuals with chronic conditions [2]. Furthermore, individualized high‐intensity loads are typically introduced progressively, once participants have adapted to exercise, in line with the fundamental principles of exercise training [3]. High‐intensity sessions are performed for limited periods, even for athletes [4], employing proper techniques and carefully controlling multiple variables to avoid musculoskeletal overload, including exercise selection, volume, range of motion, rest intervals, among others [5, 6, 7].

Notably, the fact that MBE does not involve exercise machines, elastic bands or other equipment does not imply that it is not performed at high intensities or does not increase musculoskeletal overload. For instance, frail older adults with mobility limitations and chronic conditions (e.g., osteoarthritis) may find it difficult to perform and maintain yoga poses, as they often experience muscle fatigue and weakness [8], along with the increased joint overload caused by weight‐bearing movements in this population [9]. In addition, stretching exercises can increase delayed onset muscle soreness if not properly prescribed [10].

In contrast, resistance exercises can be performed in seated or lying positions, allowing for controlled internal and external loads to target specific muscle groups [3, 11, 12], while preserving or minimizing stimulation of other body parts affected by limitations or pain. As such, it is possible that some individuals may find MBE more challenging than resistance training, possibly leading them to exercise at high intensities without proper control of key exercise variables and without prior adaptation, potentially causing greater musculoskeletal strain.

A second aspect that warrants discussion is the authors' decision to group frailty and sarcopenia together, conducting the analysis without differentiating between these two distinct conditions. There is a considerable body of evidence to support the fact that frailty and sarcopenia are distinct entities [13, 14, 15] with different associations in terms of hospitalization [16], disability or mortality [17]. As a matter of fact, sarcopenia, particularly among community‐dwelling individuals, is often considered an early stage of frailty, with some researchers recognizing it as a potential biological substrate of the frailty syndrome [18]. As such, we believe that conducting sensitivity analyses for each condition would have been appropriate, allowing for the examination of the potential influence of participants' characteristics on study outcomes.

To address this gap, we extracted and reanalyzed the data provided by Wan and colleagues [1] according to the presence of sarcopenia or frailty. Our results differ substantially from those reported by Wan and colleagues (Figure 1). When the analysis was limited to individuals with sarcopenia, MBE was not more effective than passive control in improving isometric handgrip strength (Figure 1a) nor did it promote greater improvements in Timed ‘Up and Go!’ performance compared to active control (Figure 1b). In addition, frail individuals who participated in MBE programmes did not exhibit significant gains in isometric handgrip strength relative to those in the passive control group (Figure 1c).

**FIGURE 1:**
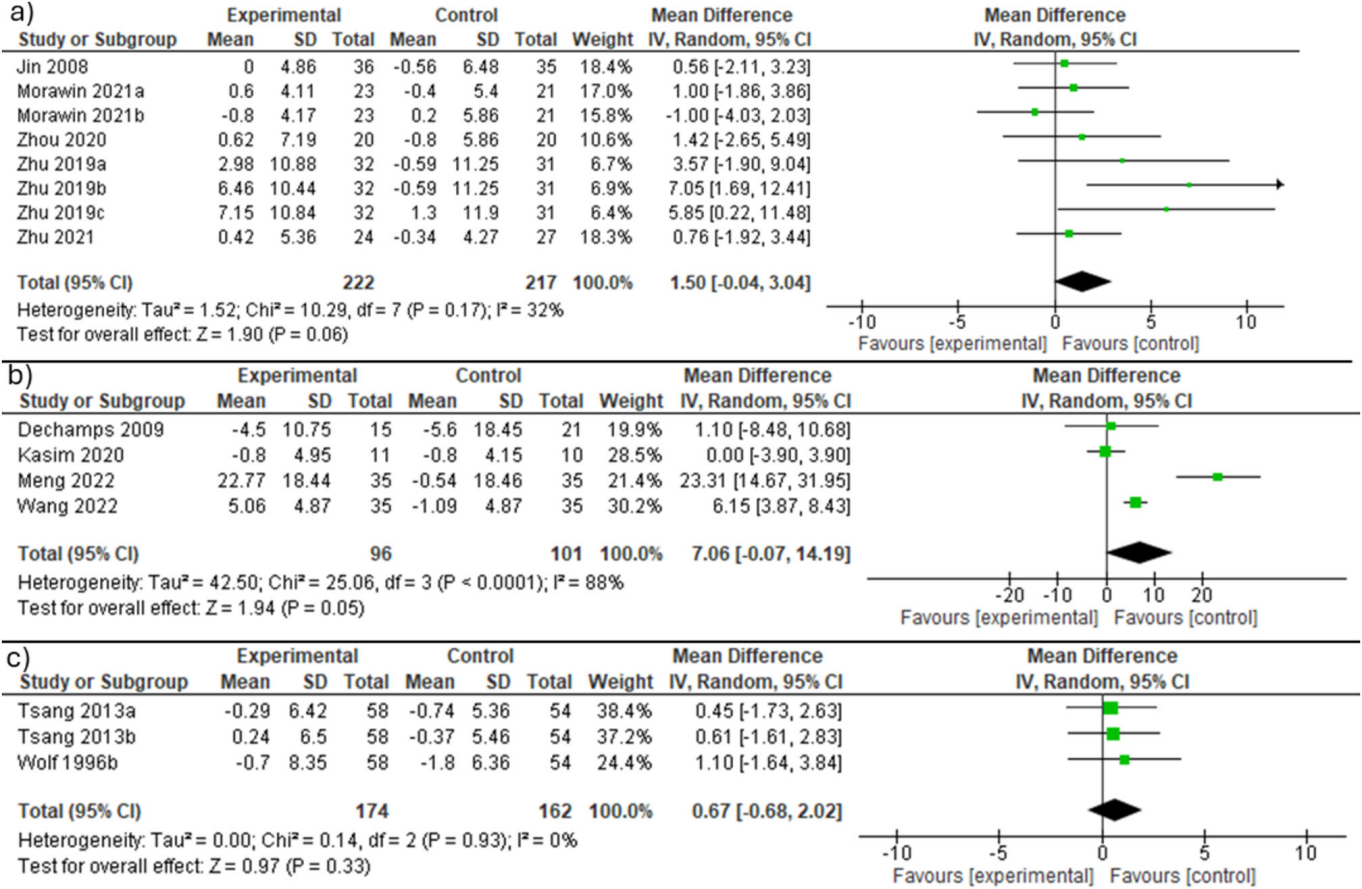
Reanalysis of the data provided by Wan and colleagues [1] on the effects of mind–body exercise on grip strength (a) and Timed ‘Up and Go’ performances (b) in individuals with sarcopenia, and grip strength (c) in those with frailty.

The next step would be to extend the stratified analysis based on the specific definitions and severity levels of the conditions studied (e.g., frailty vs. prefrailty and sarcopenia vs. severe sarcopenia) and specific components of MBE (e.g., yoga, mind‐based therapies, Tai Chi) combined with a meta‐regression to determine which variables are most strongly associated with the outcomes. However, our capacity to conduct a more detailed analysis was constrained by limited access to certain studies, despite efforts through conventional academic databases and institutional library services.

In this regard, it is important to highlight that not all the studies included in the work by Wan and colleagues are published in English. We commend the authors for not imposing a language restriction on their inclusion criteria and understand the significance of conducting the literature search in academic Chinese libraries, given the cultural and scholarly contributions of China in this field. However, the inclusion of studies that are not widely accessible could limit the replicability of the findings, which is a cornerstone of scientific validation. For example, Wan and colleagues reference a MSc dissertation (Jin et al., 2008), which may be scientifically valid. However, its inclusion requires a clear methodological statement that acknowledges the use of grey literature, along with an explanation of why the authors chose to include this type of document instead of using peer‐reviewed material only. Furthermore, we were unable to locate ‘Wang et al., 2012’ and ‘Zhang et al., 2022’ in the reference list.

Finally, in the study of Dechamps et al. [19], authors included older adults who were able to get up independently or with technical or human assistance, and who could understand basic motor instructions, while excluding terminally ill and bedridden individuals. These inclusion criteria appear vague and are not standardized methods to identify sarcopenia and frailty. Furthermore, the study by Kasim et al., which explored the effects of Tai Chi on physical and psychological aspects related to frailty, does not explicitly state that the intervention targeted frail individuals, raising concerns about its inclusion in the study by Wan et al. [1].

In conclusion, promising results emerge from original studies examining the effects of MBE on sarcopenia and frailty parameters. However, more in‐depth analyses of high‐quality randomized controlled trials are needed to provide solid evidence to establish its efficacy, safety and potential as a viable intervention for managing these conditions.

## Conflicts of Interest

The authors declare no conflicts of interest.
